# Editorial: Coumarins: New synthetic approaches and new pharmacological applications

**DOI:** 10.3389/fchem.2022.1124816

**Published:** 2023-01-06

**Authors:** Angela Stefanachi, Giovanni Muncipinto, Francesco Leonetti

**Affiliations:** ^1^ Department of Pharmacy-Pharmaceutical Sciences, University of Bari Aldo Moro, Bari, Italy; ^2^ Photys Therapeutics, Cambridge, MA, United States

**Keywords:** coumarins, syntesis, pharmacology, new methodologies, chemistry

Coumarin, a 2H-chromen-2-one oxa-heterocycle, has been extensively studied due to its occurrence in many biologically active compounds, such as Umbelliferon, the anticoagulants acenocoumarin and Warfarin, and the antibiotic Novobiocin. The ubiquity of coumarin is most likely due to its chemical structure flat, aromatic, and lipophilic with a lactone group that allows for binding to diverse targets through hydrophobic, π–π stacking, hydrogen bonds, and dipole-dipole interactions. In addition, the diversity of its substituents, which can be introduced through regioselective synthesis (also naturally occurring) on the coumarin core, accounts for the variety of pharmacological effects seen in many coumarin derivatives. Indeed, depending on the substituents and branching positions around the bicyclic core, coumarin-containing compounds have shown diverse pharmacological activities, ranging from anticoagulant activities to anti-inflammatory, antimicrobial, anti-HIV, and antitumor effects (Stefanachi et al., 2018).

The guest editors’ interest in this core has its roots in their common doctoral studies under the supervision of Prof. Angelo Carotti at the University of Bari in taly. The main scientific results of Prof Carotti’s research group’s 20-year investigation on the coumarin core, either as a scaffold or a pharmacophore moiety, have been reported in this Research Topic (Pisani et al.).

The articles included in this Research Topic illustrate how coumarins can function as highly sensitive and selective fluorophores, and also present new synthetic methods to access coumarin derivatives with biological activities.

Coumarin has frequently been used as fluorophore because of its π–π conjugated system. Wang et al. report the evaluation of the applicability of various superoxide anion sensors that were designed and synthesized using either coumarin or chromone as the fluorophores. The authors report the preference of both types of probes for various reactive oxygen species (ROS), and studies on their detection selectivity of redox-based O_2_^•−^, thus obtaining very selective redox-based O_2_^•−^ probes (Wang et al.).

Continuous attempts to develop new synthetic methods for the coumarin core have been reported, including the use of new catalysts, green reaction conditions, and the application of more recent techniques such as microwaves and ultrasounds.

Indeed, Singh et al. report a short and efficient multicomponent sequence for synthesizing fused novel polyheterocyclic chromeno spiro-pyrrolidine oxindoles via 1,3-dipolar cycloaddition reaction mediated by reactive azomethine ylides and catalyzed by the Graphene Oxide (GO). Their methodology proved to be both greener and labour-saving as the products were isolated by simple filtration without the use of any chromatographic techniques.

Furthermore, Alshabanah et al. report a three-component reaction for the synthesis of novel 3-heteroaryl-coumarin utilizing acetylcoumarin synthon under ultrasonic irradiation and chitosan-grafted poly (vinylpyridine) as an eco-friendly catalyst. This facile and efficient procedure generated compounds with a thiazole ring linked to the coumarin moiety, including three new compounds that showed promising anticancer activity against HEPG2-1 tumoral cells.

Finally, Balewski et al. describe the incorporation of a metal ion into coumarin derivatives. Accordingly, some of these new ligands have been found to display promising antioxidant, antitumor, or antibacterial activities compared to coumarin-based ligands.

In conclusion, this Research Topic shows the importance and the versatility of the coumarin core in applications of organic and medicinal chemistry.
